# Ascorbic Acid Potentiation of Arsenic Trioxide Anticancer Activity Against Acute Promyelocytic Leukemia

**DOI:** 10.1111/j.1753-5174.2009.00022.x

**Published:** 2009-12

**Authors:** Clement Yedjou, Laurette Thuisseu, Christine Tchounwou, Maria Gomes, Carolyn Howard, Paul Tchounwou

**Affiliations:** Cellomics and Toxicogenomics Research Laboratory, NIH-Center for Environmental Health, College of Science, Engineering and Technology, Jackson State UniversityJackson, MS, USA

**Keywords:** Arsenic Trioxide, Ascorbic Acid, Acute Promyelocytic Leukemia, Cell Viability, Apoptosis

## Abstract

**Introduction:**

Acute promyelocytic leukemia (APL) is a malignant disorder of the white blood cells. Arsenic trioxide (As_2_O_3_) has been used as a therapeutic agent to treat APL and other tumors. Studies suggest that ascorbic acid (AA) supplementation may improve the clinical outcome of As_2_O_3_ for APL patients. Our aim was to use human leukemia (HL-60) APL-cells as an *in vitro* test model to evaluate the effect of physiologic doses of AA on As_2_O_3_-induced toxicity and apoptosis of HL-60 cells.

**Methods:**

HL-60 cells were treated either with a pharmacologic dose of As_2_O_3_ alone and with several physiologic doses of AA. Cell survival was determined by trypan blue exclusion test. The extent of oxidative cell/tissue damage was determined by measuring lipid hydroperoxide concentration by spectrophotometry. Cell apoptosis was measured by flow cytometry using Annexin-V and propidium iodide (PI) staining.

**Results:**

AA treatment potentiates the cytotoxicity of As_2_O_3_ in HL-60 cells. Viability decreased from (58 ± 3)% in cells with As_2_O_3_ alone to (47 ± 2)% in cells treated with 100 µM AA and 6 µg/mL As_2_O_3_ with *P* < 0.05. There was a significant (*P* < 0.05) increase in lipid hydroperoxide concentrations in HL-60 cells co-treated with AA compared to As_2_O_3_ alone. Flow cytometry assessment (Annexin V FITC/PI) suggested that AA co-treatment induces more apoptosis of HL-60 cells than did As_2_O_3_ alone, but this was not statistically significant. Taken together, our experiment indicates that As_2_O_3_ induced *in vitro* cell death and apoptosis of HL-60 cells. Administration of physiologic doses of AA enhanced As_2_O_3_-induced cytotoxicity, oxidative cell/tissue damage, and apoptosis of HL-60 cells through externalization of phosphatidylserine.

**Conclusions:**

These suggest that AA may enhance the cytotoxicity of As_2_O_3_, suggesting a possible future role of AA/As_2_O_3_ combination therapy in patients with APL.

## I. Introduction

Acute promyelocytic leukemia (APL) is a malignant disorder of the white blood cells which can affect patients of all ages. Arsenic trioxide (As_2_O_3_) is been used as a therapeutic agent to treat APL [1] and other tumors [2]. In 2000, the U.S. Food and Drug Administration (FDA) approved the use of As_2_O_3_ (Trisenox) to treat relapsed APL [3]. *In vitro* studies have shown that As_2_O_3_ exerts a dual dose-dependent effect on APL cells by inducing partial differentiation at low concentrations and apoptosis at high concentrations [4,5]. Recently, we reported that the pharmacological effect of As_2_O_3_ as an effective anti-cancer drug is associated with its cytotoxic and genotoxic effects in human leukemia (HL-60) cells [6,7]. Current research in our laboratory indicates that As_2_O_3_ induces transcription of specific genes that affect mitogen response, cell cycle progression, programmed cell death, and cellular function in many ways in cultured human leukemia (HL-60) cells. Among these cellular responses to As_2_O_3_ in human leukemia (HL-60) cells are up-regulation of p53 tumor suppressor protein and repression of the *c-fos* transcription factor involved in cell cycle arrest or apoptosis, activation of *cyclin* D1 and *cyclin* A involved in cell cycle progression [8]. Other studies indicate that As_2_O_3_ induces the generation of reactive oxygen species that contribute significantly to cell killing [2,9,10] promotion of differentiation, and inhibition of growth [6].

As_2_O_3_ has also been used effectively in combination with other chemotherapeutic agents such as all-trans retinoic acid to treat APL [11]. Ascorbic acid (AA) is a natural supplement in our diet that has been studied for the prevention of human cancer and improvement of human health [12]. For many years, some scientists have claimed that use of high doses of ascorbic acid (>10 g/day) cure infections with common cold and treat AA can be used to treat cancers diseases because of the effect on the immune system [13]. Others researchers have reported that AA is effective in the prevention of cancer and protection against DNA damage through the neutralization of free radicals [14,15]. They also reported that AA may act as a pro-oxidant that helps the body's own free radical defense mechanism destroy tumors in their early stages [15,16]. Several recent studies have provided evidence that AA may extend the therapeutic spectrum of As_2_O_3_ in APL patients [17] and multiple myeloma patients [18]. However, little is known about the mechanisms of action of AA when combined with As_2_O_3_ for the treatment of APL. Therefore, the aim of this research was to use human leukemia (HL-60) APL-cells as an *in vitro* test model to determine the potential mechanism of action of AA on As_2_O_3_ chemotherapy of APL.

## II. Materials and Methods

### Chemicals and Test Media

Arsenic trioxide (As_2_O_3_), CASRN 1327-53-3, MW 197.84, with an active ingredient of 100% (w/v) arsenic in 10% nitric acid was purchased from Fisher Scientific in (Houston Texas). Growth medium RPMI 1640 containing 1 mmol/L L-glutamine was purchased from Gibco BRL products (Grand Island, NY). Fetal bovine serum (FBS), ascorbic acid, phosphate buffered saline (PBS), and MTT assay kit were obtained from Sigma Chemical Company (St. Louis, MO). Lipid peroxidation kit was purchased from Calbiochem-Novabiochem (San Diego, CA).

### Tissue Culture

Human leukemia (HL-60) APL-cells, purchased from the American Type Culture Collection -ATCC (Manassas, VA), was thawed by gentle agitation of their containers (vials) for 2 minutes in a water bath at 37°C. After thawing, the content of each vial of cell was transferred to a 25 cm^2^ tissue culture flask, diluted with up to 10mL of RPMI 1640 containing 1 mmol/L L-glutamine (GIBCO/BRL, Gaithersburg, MD) and supplemented with 10% (v/v) fetal bovine serum (FBS), and 1% (w/v) penicillin/streptomycin. The 25 cm^2^ culture flasks, each containing 2 × 10^6^ viable cells, were observed under the microscope, followed by incubation in a humidified 5% CO_2_ incubator at 37°C. Three times a week, they were diluted under same conditions to maintain a density of 5 × 10^5^/mL, and harvested in the exponential phase of growth. The cell viability was assessed by the trypan blue exclusion test (Life Technologies) and manually counted using a hemocytometer.

### Treatment and Measurement of Cell Viability

In a recently published experiment, we reported that physiologic doses of As_2_O_3_ increased cellular proliferation while pharmacologic doses of As_2_O_3_ were highly cytotoxic to HL-60 cells, showing a 24 hours LD_50_ of 6.4 ± 0.6 µg/mL [6]. Hence, to examine the effect of ascorbic acid (AA) on As_2_O_3_-induced cytotoxicity, cells exposed to physiologic doses of AA (25, 50, and 100 µM) 30 minutes prior were treated with 6 µg/mL As_2_O_3_ and incubated in humidified 5% CO_2_ incubator at 37°C for 24 hours. After the treatment period, the cell viability of human leukemia (HL-60) cells was determined by standard live-dead staining. To this end, ten µl of a 0.5% solution of the dye (trypan blue) was added to 100 µL of treated cells (1.0 × 10^5^/mL). The number of viable (transparent) and dead (blue) cells was examined on a light microscopic analysis.

### Measurement of Lipid Hydroperoxide

Lipid peroxidation is traditionally quantified by measuring malondialdehyde and 4-hydroxynonenal. These assays are nonspecific and often lead to mis-estimation of lipid peroxidation. A new lipid hydroperoxide assay kit [Calbiochem-Novabiochem, San Diego, CA] was used in this study to measure the hydroperoxide concentration by directly using the redox reactions with ferrous ions. The extraction procedure and measurement of the extracted lipid hydroperoxides was performed according to the manufacturer's instructions (Calbiochem-Novabiochem, San Diego, CA) [19,20].

Briefly, untreated and treated HL-60 cells were washed twice with cold PBS and counted for the assay. A total volume of 20 × 10^6^ cells/mL in cold PBS were mixed with 3 mL of chloroform/methanol (2:1) solution, vortexed and mixed well. The sample was centrifuged at 1000 × *g* for 5 minutes at 0°C until phase separation was achieved. The pasteur pipet was used carefully to collect 700 µL of chloroform layer in the bottom of the test tube and transferred to another test tube. Freshly prepared chromogen (50 µL) was added to each test tube, vortexed and mixed well. The sample was incubated at room temperature for 5 minutes. The absorbance of the sample was monitored at 500 nm, and the concentration of lipid hydroperoxide was determined from a standard curve.

### Annexin V FITC/PI Binding Assay by Flow Cytometry

The response of HL-60 cells to arsenic trioxide (As_2_O_3_) alone and ascorbic acid (AA) plus As_2_O_3_ was assessed by flow cytometry using Annexin V FITC/PI staining kit. After 24 hours of exposure to either a pharmacologic dose of As_2_O_3_, or different physiologic doses of AA plus a pharmacologic dose of As_2_O_3_, 1 × 10^6^ cells/mL were washed in PBS, re-suspended in binding buffer (10 mm Hepes/NaOH pH 7.4, 140 mm NaCl, 2.5 mM CaCl_2_), and stained with FITC-conjugated annexin V (Pharmingen, Becton Dickinson Co., San Diego, CA, USA). Then, cells were incubated for 15 minutes in the dark at room temperature, washed with binding buffer and analysed by flow cytometry (FACS Calibar; Becton-Dickinson) using CellQuest software.

### Statistical Analysis

Experiments were performed at least in triplicates. Data were represented as means ± SDs. Where appropriate, one-way anova test or Student paired t-test was performed using SAS Software available in the Bio-statistics Core Laboratory at Jackson State University. *P* values less than 0.05 were considered statistically significant.

## III. Results

### Ascorbic Acid Enhances the Cytotoxicity of Arsenic Trioxide in HL-60 Cells

**Figure 1:**
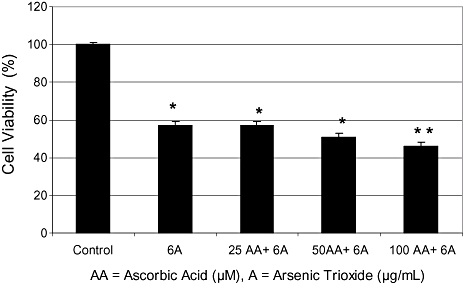
Potential effect of co-administration of ascorbic acid (AA) and arsenic trioxide (As_2_O_3_) to human leukemia (HL-60) cells. HL-60 cells were cultured in the absence or presence of AA and As_2_O_3_ or in combination of AA and As_2_O_3_ for 24 hr as indicated in the Materials and Methods. Cell viability was determined based on the trypan blue exclusion test. Each point represents a mean value and standard deviation of 3 experiments with 6 replicates per dose. *Significantly different from the control by anova Dunnett's test; *P* < 0.05. **Significantly different from As_2_O_3_ alone by anova Dunnett's test; *P* < 0.05.

In the present study, HL-60 cells were treated either with a pharmacologic dose of As_2_O_3_, or with various physiologic doses of AA plus As_2_O_3_ as described in the trypan blue exclusion test. As shown in (Figure 1), As_2_O_3_ is highly cytotoxic to HL-60 cells at 6 µg/mL of exposure. Co-treatment of these cells using physiologic concentrations (25–100 µM) of AA and a pharmacologic dose (6 µg/mL) of As_2_O_3_ resulted in a higher level of cell death than did As_2_O_3_ alone. We found that the viability of HL-60 cells declined from (58 ± 3)% in cells treated with As_2_O_3_ alone to (47 ± 2)% in cells treated with 100 µM AA and 6 µg/mL As_2_O_3_ with *P* < 0.05.

### Ascorbic Acid Enhances Lipid Hydroperoxide Generation in Arsenic Trioxide-treated HL-60 Cells

**Figure 2:**
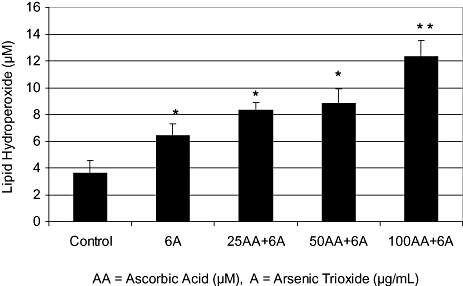
AA potentiation on As_2_O_3_-induced oxidative stress in HL-60 cells. Cells were incubated for 24 hr with 6 µg/mL As_2_O_3_ and various concentrations of AA (25, 50, and 100 µM). Lipid hydroperoxide formation was determined as described in Materials and Methods. *Significantly different from the control by anova Dunnett's test; *P* < 0.05. **Significantly different from As_2_O_3_ alone by anova Dunnett's test; *P* < 0.05. Data are representative of 3 independent experiments.

A high level of lipid hydroperoxide concentration was detected in HL-60 cells after 24 hours of As_2_O_3_ treatment compared to control cells (Figure 2). A concentration-dependent increase in lipid hydroperoxide generation was observed in HL-60 cells co-treated with ascorbic acid (AA) and As_2_O_3_ compared to As_2_O_3_ alone. Taken together, co-administration of AA and As_2_O_3_ in culture cells caused significant (*P* < 0.05) increase of lipid hydroperoxide concentration resulting from oxidation of fatty acids and/or degradation products of poly-unsaturated fatty acids. Findings from this experiment suggest that the pro-oxidant property of AA *in vitro* may increase reactive oxygen species (ROS) formation that potentiates the cytotoxicity of As_2_O_3_.

### Ascorbic Acid Enhances Arsenic Trioxide-induced Apoptosis in HL-60 Cells

**Figure 3:**
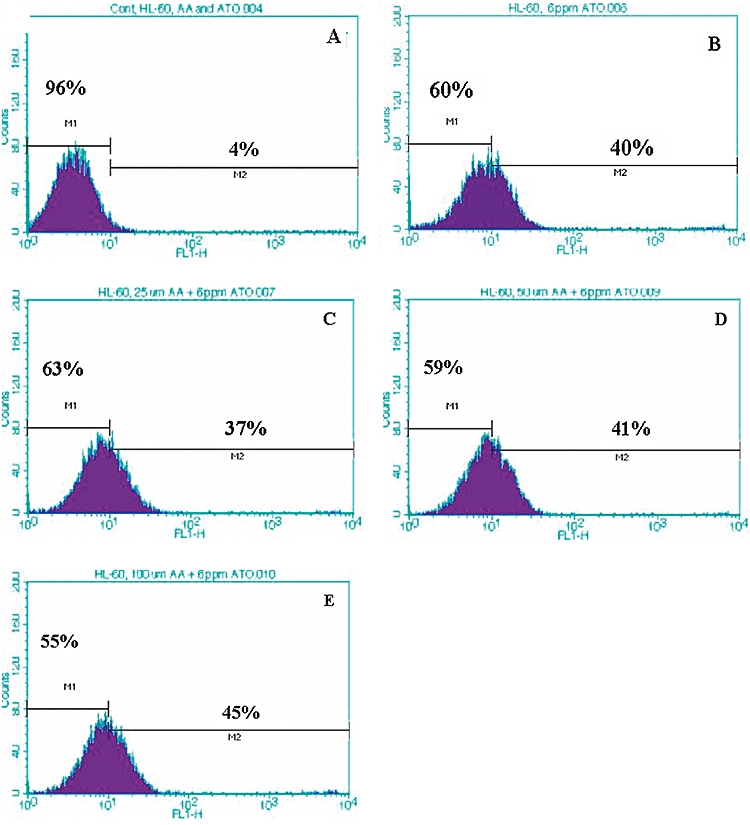
Representative flow cytometry analysis data from Annexin V-FITC staining. The histogram shows a comparison of the distribution of annexin V negative cells (M1) and annexin V positive cells (M2) after 24 h incubation in HL-60 cells. A: control; B: 6 µg/mL As_2_O_3_; C: 25 µM AA + 6 µg/mL As_2_O_3_; D: 50 µM AA + 6 µg/mL As_2_O_3_; E: 100 µM AA + 6 µg/mL As_2_O_3_.

**Figure 4:**
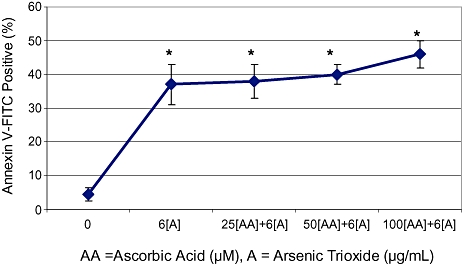
Annexin V-FITC positive cells induced by either arsenic trioxide alone or ascorbic acid and arsenic trioxide combination in HL-60 cells. Each point represents the mean value and the standard deviation of three experiments, showing similar results. *Significantly different from control (0 µg/mL), *P* < 0.05.

To determine whether physiologic doses of ascorbic acid (AA) could sensitize arsenic trioxide (As_2_O_3_)-mediated apoptosis, HL-60 cells were treated for 24 hours, subsequently stained with annexin V/PI, and analyzed by flow cytometry. As shown in (Figures 3 and 4), AA enhanced the percentage of cells positive for annexin V, but this was not statistical significant. The percentage of cells positive for annexin V was (40 ± 5)% in cells treated with As_2_O_3_ alone and (46 ± 4)% in those treated with 100 µM AA and 6 µg/mL As_2_O_3_ with *P* > 0.05.

## IV. Discussion

### Ascorbic Acid Enhances the Cytotoxicity of Arsenic Trioxide in HL-60 Cells

In this study, we investigated the cellular effect of ascorbic acid (AA) in conjunction with arsenic trioxide (As_2_O_3_) in human leukemia (HL-60) cells. Our data showed increased cell death in the human leukemia (HL-60) cells at physiologic doses (25–100 µM) of AA and pharmacologic dose (6 µg/mL) of As_2_O_3_, indicating potentiation effect between AA and As_2_O_3_. We found that the combination of physiologic doses of AA and pharmacologic dose of As_2_O_3_ is more cytotoxic to HL-60 cells compared to As_2_O_3_ alone. We previously demonstrated that low or physiologic doses (25–100 µM) of AA were not cytotoxic, suggesting that AA has the potential to be safe and acts as effective chemosensitizing agent in As_2_O_3_-based chemotherapy [21]. Zhang and his co-workers have reported the role of physiologic doses of AA in gastric cancer cells [22]. Preclinical studies have shown the efficacy of As_2_O_3_ on various cultured human (HL-60, HepG_2_, Jurkat) cancer cell lines [7]. Current research has reported many possible treatments for APL patients [23,24]. However, a common treatment remains As_2_O_3_, or perhaps a combination of AA and As_2_O_3_[18]. Interestingly, finding from our present studies suggest that the combination of these two compounds could be a more proficient treatment in killing cancer cells compared to As_2_O_3_ alone. Similar to our findings, previous studies have shown that AA potentiates As_2_O_3_-mediated cytotoxicity in U266 and multiple myeloma cells [18,25]. The use of AA alone has a controversial history in cancer treatment [26]. Cameron and Pauling reported that AA or ascorbate, given in pharmacologic doses of 10 g/day, is effective in treating some cancers and improving patient well-being [12]. One the contrary, Moertel and his co-workers reported that the same dose of AA had no effect on patient well-being and survival in two double-blind placebo-controlled trials [27].

### Ascorbic Acid Enhances Lipid Hydroperoxide Generation in Arsenic Trioxide-treated HL-60 Cells

To investigate the hypothesis that ascorbic acid (AA) enhances lipid hydroperoxide generation in arsenic trioxide (As_2_O_3_)-treated cells. HL-60 cells were exposed to different physiologic doses (25–100 µM) of AA and a pharmacologic dose (6 µg/mL) of As_2_O_3_ for 24 hours. Our results indicate that the treatment of HL-60 cells with As_2_O_3_ alone produces a significantly higher level of lipid hydroperoxide, a major mediator of oxidative stress and cellular injury that often leads to cell death. This significant increase in lipid hydroperoxide concentrations was further exacerbated by AA co-treatment. Based on these results, it is evident that AA acts as a pro-oxidant in As_2_O_3_-treated HL-60 cells. Our findings are in agreement with a previous report indicating that AA exhibits pro-oxidant activity in the presence of free transition metals [28]. The relatively higher sensitivity of tumor cells to the pro-oxidant action of AA may be related to its lower antioxidant defense and to the presence of transition metals [29,30]. On the contrary, AA has an antioxidant effect in the absence of metals, but becomes a pro-oxidant when they are present [31,32]. Because AA potentiated As_2_O_3_-mediated cell death, it is possible that As_2_O_3_ treatment increased reactive oxygen species (ROS) production. Consistent with this finding, published reports indicate that arsenic induces the generation of reactive oxygen species (ROS) that contribute significantly to cell killing [10,33]. Another study indicates that the cytotoxic and genotoxic effects of As_2_O_3_ are mediated through oxidative stress [7].

### Ascorbic Acid Enhances Arsenic Trioxide-induced Apoptosis in HL-60 Cells

Annexin-V is a specific phosphatidylserine-binding protein used to detect apoptotic cells by providing an assessment of the progression from living cells (annexin−/PI−) towards apoptotic stage (annexin+/PI−) and postapoptotic cell death (annexin+/PI+). Our data show a progressive non-significant increase of apoptotic cells which reach the highest value in the presence of AA and As_2_O_3_ (Figures 3 and 4). This finding was not statistically significant, but a limitation of the study was the small number of experiments performed. Recent literature has indicated that low concentrations of As_2_O_3_ (2 µM) induces apoptosis in HPV 16 DNA-immortalized human cervical epithelial cells and that its molecular pathways leading to apoptosis may be associated with down-regulation of viral oncogene expression [34]. Others have reported that As_2_O_3_ selectively induces acute promyelocytic leukemia cell apoptosis via a hydrogen peroxide-dependent pathway (10). Using the trypan blue exclusion test and the flow cytometry analysis, we have shown in the present study that As_2_O_3_ causes substantial cell death and apoptosis of HL-60 cells. Administration of physiologic doses of AA was sufficient to enhance As_2_O_3_-induced cytotoxicity, oxidative cell/tissue damage, and apoptosis of HL-60 cells. These findings highlight the potential effect of AA in promoting the pharmacologic effect of As_2_O_3_, suggesting a possible future role of AA/As_2_O_3_ combination therapy in patients with APL.

## Conclusions

Ascorbic acid (AA) and arsenic trioxide (As_2_O_3_) co-treatment exerts dual effects on human leukemia (HL-60) cells by inducing oxidative stress and subsequent inhibition of cell growth and induction of apoptosis. The trypan blue exclusion test results indicated that AA and As_2_O_3_ combination significantly (*P* < 0.05) reduced cell viability of human leukemia (HL-60) cells stronger than did As_2_O_3_ alone. Although the mechanism by which AA enhances As_2_O_3_-mediated cytotoxicity in HL-60 cells remains unknown, here we provide evidence that AA potentiates As_2_O_3_-induced toxicity through oxidative cell/tissue damage and perhaps via apoptosis in human leukemia (HL-60) cells. Based on this knowledge, the elucidation of the synergy and mechanisms of action between AA/As_2_O_3_ may eventually lead to a more effective approach for the management of patients with APL.
